# Organ-sparing radiotherapy in penile cancer: therapeutic approaches

**DOI:** 10.1007/s11255-026-05135-y

**Published:** 2026-04-17

**Authors:** Avraam Muresan, Mihai Domnutiu Suciu, Vlad Horia Schitcu, Victor Ona, Norberth-Istvan Varga, Cristina Maria Muresan, Gabriel Kacso

**Affiliations:** 1 Doctoral School, “Iuliu Haţieganu” University of Medicine and Pharmacy, Victor Babeş Street, No. 8, 400012 Cluj-Napoca, Romania; 2 Institute of Urology and Renal Transplantation Cluj-Napoca, 4–6 Clinicilor Street, 400006 Cluj-Napoca, Romania; 3 Prof. Dr. Ion Chiricuță Oncology Institute, 34–36 Republicii Street, 400015 Cluj-Napoca, Romania; 4 Municipal Clinical Hospital Cluj-Napoca, 11–13 B.P. Hașdeu Street, 400371 Cluj-Napoca, Romania; 5 Doctoral School, “Victor Babes” University of Medicine and Pharmacy, Eftimie Murgu Square No. 2, 300041 Timisoara, Romania; 6 Cluj County Emergency Clinical Hospital, 3–5 Clinicilor Street, 400006 Cluj-Napoca, Romania; 7 Amethyst Radiotherapy Center Cluj, 102 Frunzișului Street, 400664 Cluj-Napoca, Romania

**Keywords:** Penile cancer, Brachytherapy, Low-dose-rate brachytherapy, Pulsed-dose-rate brachytherapy, High-dose-rate brachytherapy, Organ preservation

## Abstract

**Background/Objective:**

The primary treatment for penile squamous cell carcinoma (PSCC) is often partial or total penectomy, which causes significant functional morbidity and psychological distress. Brachytherapy (BT) has emerged as a primary, organ-preserving alternative. The objective of this systematic review was to analyze the oncologic efficacy, organ preservation rates, and functional outcomes of LDR, PDR, and HDR brachytherapy.

**Methods:**

This systematic review (PROSPERO: CRD420251136499) followed PRISMA guidelines. We searched PubMed, Scopus, ScienceDirect, and Google Scholar (to August 2025) for studies (*n* ≥ 10) reporting clinical outcomes of LDR, PDR, or HDR brachytherapy for adult penile cancer. Risk of bias was assessed using NIH tools and the Cochrane RoB tool. Due to high heterogeneity in study design and treatment techniques, a quantitative synthesis (narrative) was performed.

**Results:**

Eighteen studies were included. Five-year cancer-specific survival (CSS) ranged from 85.0 to 100% across 9 studies that explicitly reported this metric. Local failures, when they occurred, were demonstrated to be highly salvageable (77–100% success).

**Conclusions:**

The evidence, though limited and highly heterogeneous, confirms that penile brachytherapy is an effective, curative-intent, organ-sparing treatment for T1–T2 disease.

**Supplementary Information:**

The online version contains supplementary material available at 10.1007/s11255-026-05135-y.

## Introduction

Penile cancer is an uncommon malignancy, representing less than 1% of all male cancers in developed countries, with an estimated incidence of approximately 1 per 100,000 men annually in Europe and North America [1–5]. However, its incidence is substantially higher in parts of South America, Africa, and South Asia, where penile carcinoma can account for up to 10% of all male malignancies [6]. The global variation in disease burden reflects differences in and the importance of socioeconomic conditions, hygiene, circumcision practices, and, importantly, the prevalence of human papillomavirus (HPV) infection [7].

Historically, surgical excision—ranging from wide local excision to partial or total penectomy—has been the standard of care for localized disease [8]. Although these approaches provide local control by their radical nature, they often result in significant functional and psychosocial morbidity, including impaired sexual function, altered body image, and reduced quality of life [9–12]. To minimize these effects, penile-sparing surgical techniques, such as glans resurfacing, wide local excision with grafting, laser ablation, and glansectomy, have been introduced. Applicability depends on tumor extent, anatomical constraints, and the availability of surgical expertise. Even when feasible, the conservative surgical approach may present unsatisfying local recurrence [13, 14].

Radiotherapy (RT) is an organ-preserving treatment option for penile cancer and may achieve comparable outcomes to surgery in selected patients. Despite its long-standing use, RT for penile cancer remains underutilized, partly due to limited expertise, lack of standardization, and the rarity of the disease [15, 16]. Recent advances in imaging, treatment planning, and dose delivery have strengthened organ-sparing radiotherapy approaches [15, 17, 18]. These advances include three-dimensional image guidance, MRI-based contouring, and adaptive planning, improving precision, reproducibility, and sparing of organs at risk (OARs) [19–22].

Among RT modalities, brachytherapy (BT) involves direct placement of radioactive sources within or adjacent to the tumor, providing steep dose gradients and high conformality. Historically, low-dose-rate (LDR) brachytherapy using iridium or caesium sources was the standard, while pulsed-dose-rate (PDR) and high-dose-rate (HDR) after loading systems have emerged as modern adaptations offering enhanced dosimetry precision, radioprotection, and procedural control [23]. This facilitates delivery of tumouricidal doses while sparing adjacent healthy tissue, such as urethra, glans, and corpora cavernosa.

Low-dose-rate (LDR), pulsed-dose-rate (PDR), and high-dose-rate (HDR) brachytherapy are differentiated by their dose delivery rates and radiobiological characteristics. LDR brachytherapy delivers radiation continuously at approximately 0.4–2 Gy per hour using low-activity sources, allowing sublethal DNA repair in normal tissues during prolonged exposure. PDR brachytherapy utilises a high-activity source to administer hourly pulses of radiation designed to reproduce the biological effects of LDR, assuming comparable tissue repair kinetics [24, 25]. HDR brachytherapy employs the same radionuclide at higher activity levels to deliver doses greater than 12 Gy per hour in brief, fractionated sessions lasting several minutes. Differences in dose-rate structure result in distinct cellular responses, where LDR and PDR permit continuous or intermittent repair of sublethal damage, and HDR produces concentrated DNA double-strand breaks that require careful dose fractionation to maintain normal tissue tolerance [26].

Evidence from other HPV-related squamous cell carcinomas (oropharyngeal, anal, and cervical) suggests that HPV-associated tumors may be more radiosensitive, supporting the hypothesis that similar biology could influence radiotherapy tailoring in penile cancer [27–29]. It has been described that HPV DNA is detected in around one-third of penile carcinomas, predominantly genotypes 16 and 18, which have been associated with improved prognosis and increased radiosensitivity [30–32]. In terms of histological characteristics, the predominant subtype is squamous cell carcinoma (SCC), comprising approximately 95% of cases [33–35]. These characteristics provide optimistic prospects for organ-sparing radiotherapy since radiotherapy has long been used as the standard approach for various localizations of squamous cell carcinoma when aiming for organ-sparing objectives [36–38].

The scattered literature motivated the idea of systematic evidence synthesis, to clarify the optimal role, technique, and outcomes of radiotherapy in this setting. Current work builds upon a previous wider synthesis which analyzed wider options for treatment approaches in penile cancer [39–41]. Timeframe and scope of previous work leave space for more up to date, targeted research.

In the described context, this systematic review aims to provide a critical synthesis and appraisal of the existing evidence regarding low-dose-rate (LDR), pulsed-dose-rate (PDR), and high-dose-rate (HDR) brachytherapy as organ-sparing treatments for penile squamous cell carcinoma. Specifically, we address a clinically important knowledge gap: while brachytherapy has been used for decades, the comparative evidence across dose-rate modalities remains scattered and unstandardized, preventing evidence-based selection between LDR, PDR, and HDR approaches. This review systematically determines whether variations in dose-rate technique are associated with differences in oncologic efficacy, complication rates, or preservation of sexual and urinary function, and critically evaluates the strength and consistency of current clinical evidence to guide treatment selection.

## Materials and methods

### Guideline and PICO

This systematic review was conducted in accordance with the Preferred Reporting Items for Systematic Reviews and Meta-Analyses (PRISMA) 2020 statement [42]. The completed PRISMA checklist is available in the Supplementary Materials (Table S1). The review protocol was prospectively registered in the PROSPERO International Prospective Register of Systematic Reviews (registration number: CRD420251136499).

To ensure methodological rigor, the review question was structured using the PICO framework (Population, Intervention, Comparator, and Outcomes), defined as follows:

- Population (P): Adult patients (≥ 18 years) diagnosed with penile cancer (any stage, including squamous cell carcinoma or other histological sub-types) treated with organ-sparing brachytherapy.
- Intervention (I): Organ-sparing brachytherapy, including high-dose-rate (HDR) and low-dose-rate (LDR) modalities.
- Comparator (C): Standard of care, alternative radiation regimens (e.g., HDR vs. LDR), or direct comparisons between HDR and LDR.
- Outcomes (O): Oncological outcomes (local control, recurrence, survival); functional outcomes (organ preservation, sexual and urinary function); treatment-related toxicity (acute and late complications); and, where reported, patient-reported outcomes (quality of life).

In this systematic review, AI was used to assist in exploring potential gaps or overlooked perspectives after the original hypothesis and objective of this systematic review were established. Specifically, the large language model Gemini (Google, Mountain View, CA, USA, 2025) was prompted to generate the search queries used in the database search and for language editing. AI’s role was limited to search-query generation and language editing and did not contribute to study selection, data extraction, analysis, or interpretation.

### Search strategy

A systematic literature search was conducted across PubMed, Scopus, ScienceDirect and Google Scholar, covering the period from 1 January 2010 to 31 August 2025. The search strategy combined controlled vocabulary (e.g., MeSH and Emtree terms) and free-text keywords related to penile cancer and brachytherapy, with Boolean operators (AND, OR) applied to refine the results. For example, the ScienceDirect database search string was restricted by the 8 Boolean operators’ limitation and looked as follows: (“penile cancer” AND brachytherapy AND (“low-dose-rate” OR "high dose rate" OR "pulsed dose rate")). Database-specific adaptations of this strategy were applied to Scopus and PubMed respectively. Exact database-specific search queries are available in Supplementary Material.

To further complement the database searches (limited by factors, such as Boolean operator caps, differences in field tagging, syntax between databases, etc.), reference lists of all included articles were manually screened, and citation tracking (“snowballing”) was performed to identify additional eligible studies.

### Selection of articles

To ensure the quality and reliability of study selection, all records were independently screened for eligibility by two reviewers (M.C.M. and M.D.S.). Inter-rater agreement, assessed using Cohen’s Kappa, was 0.84, indicating a high level of concordance. Any discrepancies were resolved through discussion, and when consensus could not be reached, a third reviewer (A.M.) was consulted.

### Inclusion criteria

Articles were selected based on the following criteria:

1. Studies including adult patients (≥ 18 years) diagnosed with penile cancer of any stage, including squamous cell carcinoma or other histological sub-types.
2. Studies in which patients were treated with organ-sparing brachytherapy, specifically high-dose-rate (HDR), pulsed-dose-rate (PDR), or low-dose-rate (LDR) techniques.
3. Studies comparing brachytherapy with alternative treatment approaches, different brachytherapy modalities (e.g., HDR vs. LDR), or the standard of care. Studies without comparators were also eligible if they reported relevant clinical outcomes.
4. Studies reporting clinical outcomes, including oncological outcomes (local control, recurrence, survival), functional outcomes (organ preservation, urinary or sexual function), treatment-related toxicity (acute or late complications), or patient-reported quality of life.
5. Studies involving human participants with sufficient methodological detail to allow assessment of patient selection and outcome reporting.
6. Peer-reviewed articles published in English, regardless of study design, including randomised controlled trials, prospective and retrospective cohorts, case–control studies, and case series with ≥ 10 patients.

### Exclusion criteria

The exclusion criteria consisted of the following:

1. Studies exclusively involving pediatric patients (< 18 years).
2. Studies not involving brachytherapy (e.g., external beam radiotherapy, surgical interventions alone, or other non-brachytherapy modalities).
3. Studies not reporting relevant clinical outcomes, such as those focusing solely on dosimetry, physics, or technical planning aspects without clinical correlation.
4. Case reports, case series with fewer than 10 patients, conference abstracts without full text, reviews, editorials, letters, or other non-original research formats.
5. Non-peer-reviewed sources or unpublished studies (e.g., opinion pieces, pre-prints, blog posts).
6. Publications with unsuitable formats, such as letters, editorials, conference abstracts, systematic reviews, or meta-analyses.
7. Articles published in languages other than English or without accessible full-text versions.

After finalizing the list of eligible studies, two independent reviewers (V.H.S. and N.I.V.) extracted data using a standardized collection. Extracted variables included study identifier (first author and year), design, sample size, patient characteristics, intervention details, comparator(s), and reported outcomes. Any missing or unclear information was documented as a limitation during critical appraisal. Studies were included in each synthesis according to the availability of outcome data, and given the heterogeneity in reporting, no data were imputed or statistically adjusted; analyses were based solely on the information provided in the original publications.

Due to the lack of RCTs and the high percentage of retrospective studies (~ 90%), quantitative data analysis was inappropriate, resulting in the qualitative synthesis approach with consideration for available data and its heterogeneity.

### Quality and risk of bias assessment

The methodological quality of the included studies was assessed using the NIH Study Quality Assessment Tools (www.nhlbi.nih.gov/health-topics/study-quality-assessment-tools; accessed 1 September 2025) by two independent reviewers (M.C.M. and M.D.S.), with any disagreements resolved through consultation with a third reviewer (A.M.). Risk of bias was assessed in the same manner, following standardized reviewer training, and using the Cochrane Collaboration’s risk of bias tool, ROBINS-I (version 2, Creative Commons Attribution-Non-Commercial-No Derivatives 4.0 International License, 2024) instrument for non-randomized studies. Risk-of-bias visualizations, including traffic-light and summary plots, were generated using the robvis R package (version 0.3.0, 2019; McGuinness & Higgins, 2020). Full study ratings and visual outputs are provided in the Supplementary Materials.

## Results

### Overview of study selection and included articles general characteristics

A total of 483 records were identified through database searches, including Scopus (*n* = 219), ScienceDirect (*n* = 173), Google Scholar (*n* = 61) and PubMed (*n* = 30). After removing 340 records through automated filtering (based on timeframe restrictions, publication type, and other such filters available on each database), 51 duplicate entries were removed. This resulted in the screening of 92 titles and abstracts.

Of these, 70 were excluded for the following reasons: unrelated exposure or outcome (*n* = 56), irrelevant population (*n* = 10) and language (besides previous filtering, *n* = 4). These 22 full-text articles were retrieved and assessed for eligibility. After excluding 6 more studies for irrelevant exposure or outcome, along with one other study with a wrong design, 15 studies were ultimately included from the database search.

**Fig. 1:**
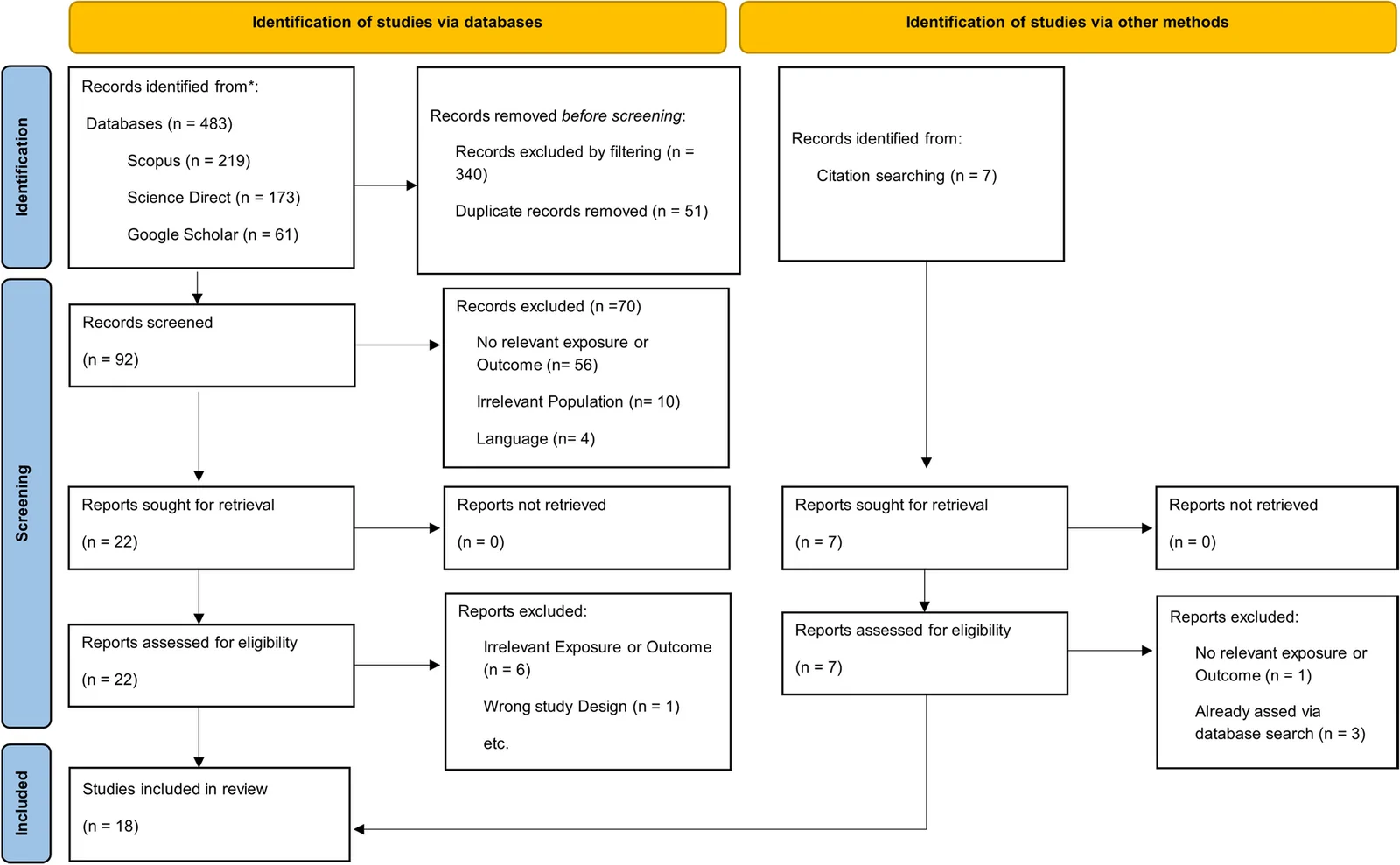
PRISMA flow diagram of the study selection process (PRISMA = Preferred Reporting Items for Systematic Reviews and Meta-analyses)

An additional 7 studies were identified via citation searching. Of these, 3 studies met the inclusion criteria after full-text review, while the rest were excluded due to prior inclusion (*n* = 3) or irrelevant outcomes (*n* = 1). Finally, the review included 18 studies (*n* = 15 + 3), as summarized in Fig. 1.

Methodological differences across the studies represented the main challenge of synthesizing and interpreting results. The main characteristics of the included studies can be found summarized in Table 1.

**Table 1:** Overview of the included studies

Author (year)	Country	Study design	Number of patients	Intervention	RFR at 5 years, % (*n*)	RFR at 1 and 3 year, (*n*)	Sexual functions core (tool name/score)	Urinary function score	Complications	PPR	Endpoints Reported	References
Makarewicz (2010)	Poland	Retrospective cohort, single institution	33	HDR & PDR	79	NR	NR (authors note erections usually maintained)	NR	Sterile distal urethritis 30%, telangiectasia 15%, soft-tissue necrosis 9%	~ 85	Organ-sparing interstitial BT achieved acceptable 5 year control and high preservation; most local failures were surgically salvaged	[43]
Petera (2011)	Czech Republic	Case series	10	HDR	NR	NR	NR	NR	Acute: edema, G2 mucositis Late: mild telangiectasia only, no necrosis or urethral stenosis	100	Early Czech single-center HDR series shows excellent short-term control and tolerance with 18 × 3 Gy BID; supports feasibility of HDR for selected early lesions	[44]
Delaunay (2013)	France/ Spain	Multicentric retrospective cohort	47	LDR	DFS 84, CSS 88 (5 year)	NR	IIEF-5, BASIC IDEA questionnaire; 59% sexually active post-BT; 94% maintained erections	NR	Ulceration 21%, meatal stenosis 21%, pain 26% (mostly mild/moderate)	66	LDR BT provided durable local control with preserved erections (94%); 58.8% sexually active; minimal psychosocial impact	[45]
Rouscoff (2014)	France	Retrospective cohort, single institution	12	HDR	83	2–3 year: 83	IIEF-5 median 16 (pre) vs 13 (post); no significant decline	IPSS unchanged	Acute dermatitis G1-2 91%, G3 8%, telangiectasia 33%, 1 G3 meatal stenosis (9%),	92	HDR series achieved strong short-term control with preserved function; feasibility and safety comparable to LDR/PDR with shorter treatment time	[46]
Sharma (2014)	India	Prospective case series	14	HDR	–	3 years: ~ 86	IIEF-5: 10/11 maintained score > 21 post-BT	NR	100% G3 skin toxicity, fibrosis 29%, no necrosis/stenosis observed	93	HDR interstitial BT showed excellent 3 year control and preservation with manageable acute toxicity; sexual function mostly maintained	[47]
Kamsu-Kom (2015)	France	Retrospective cohort, single institution	27	PDR	–	3 years: 88	NA	NR	Late ulceration 9%, urethral stenosis 22% (requiring dilatation)	85	PDR BT achieved local control and penile preservation comparable to historical LDR data, with manageable toxicity; suitable as a first-line option	[48]
Pimenta (2015)	Spain	Retrospective cohort, single institution	25	LDR & PDR & HDR	91	100 LC after 9.2 year (median FU; 2 failures)	NR	NR	Telangiectasia 48%, urethral stenosis 43%, atrophy 17%, fibrosis 9%, necrosis 4%, urinary incontinence 13%; two partial penectomies for fibrosis	86 (5 yr)	LDR/PDR BT achieved excellent long-term control and preservation in early SCC; urethral stenosis common; supports BT as standard organ-sparing therapy	[49]
Cordoba (2016)	France	Retrospective cohort, single institution	73	LDR	LC: 88	LC 1 year: 98	NR	NR	Dermatitis 12%, urinary 6%, urethral stenosis 7%, sexual pain 2%, necrosis 7%	88 (5 yr)	LDR BT achieved durable control and preservation with acceptable late toxicity; lymph node-positive and diabetic patients had poorer outcomes	[50]
Seibold (2016)	Germany	Retrospective cohort, single institution	13	PDR	10-yr local cumulative recurrence rate 12.5	DFS: 81; CSS: 78 (10 yr)	IIEF: 7/11 none–mild ED; 4/13 severe ED	NS	Necrosis 31% (medically managed), urethral stenosis 15%, dysuria 31%, telangiectasia 23%	100	PDR BT achieved durable local control and functional preservation with acceptable late toxicity; majority retained erectile function	[51]
Escande (2017)	France	Retrospective cohort, single institution	201	LDR & PDR	82	1 year 90, 10 year 72	67% sexually active; mean QoL score 75/100 (*n* = 21)	NR	Painful ulceration 21% (G2–3), meatal stenosis 26%, fibrosis/telangiectasia 30%, G3 toxicity 7%	85	Large single-center series confirms durable 5 year LC (82%) and PPR (85%) with manageable morbidity; higher dose > 62 Gy improved LC	[52]
Gambachidze (2017)	France	Retrospective cohort, single institution	23	LDR & PDR	NR	NR	IIEF-5 median = 20 (mild ED); IMGI = 21; 70% sexually active	LUTS = 6; Pilling = 2	Painful ulceration 26%, self-catheterization 30%, urethral dilation 13%, necrosis 26%	NS	LDR/PDR BT had moderate impact on urinary/sexual function with preserved QoL; majority sexually active and satisfied with appearance	[53]
Kellas-Ślęczka (2019)	Poland	Retrospective cohort, single institution	76	HDR	LC: 66	1 year: 85, 3 year: 72	NA	NR	Pigmentation 36%, telangiectasia 21%, ulceration 3%, atrophy ~ 9%, urethral stenosis 1.3% (T3 case); no G3–G4 toxicity	70 (5 year)	HDR BT achieved long-term control and preservation comparable to LDR/PDR with minimal severe toxicity; appropriate for early/selected T3 lesions	[54]
Marbán-Orejas (2020)	Canada	Retrospective cohort, single institution	15	HDR	NR	100 DFS (1 relapse per each group)	82% potency preserved (self-reported)	NR	Interstitial: necrosis 43%, stenosis 43%; mold: telangiectasia & pigmentation early cases; all G3 acute dermatitis resolved by 4 mo	86–88	Both HDR techniques achieved excellent control and preservation; recommends standardizing urethral contouring and dosimetry reporting	[55]
Garisto (2021)	Canada	Retrospective cohort, single institution	51	LDR vs PP	BT: 80 LC vs PP: 100	80	NR	NR	BT: urethral stenosis 16%, necrosis 8%, infection 2%	69	Partial penectomy yielded higher LC, but LDR BT achieved ~ 70% preservation with acceptable toxicity; salvage surgery effective	[56]
Martz (2021)	France	Retrospective cohort, single institution	29	HDR	5 year LRFS 82	5 year DFS 57	IIEF-5: no significant change vs baseline; 17% mild ED	IPSS unchanged	Acute G2 radiodermatitis 83%, G3 skin necrosis 10%, telangiectasia 17%, stenosis 7% (dilatation)	79	HDR interstitial BT achieved strong 5 year control and preservation with stable function; dose optimization essential to reduce necrosis	[57]
Pohanková (2021)	Czech Republic	Prospective cohort, single institution	31	HDR	5 year 81	1 year 100	85% maintained active sexual life (verbal assessment)	NR	Acute G2 mucositis 100%, G1 telangiectasia 23%, urethral stenosis 7%, necrosis 3%, one partial penectomy	81 (5 year); 62 (10 year)	HDR BT achieved long-term control and preservation comparable to LDR; minimal necrosis (3%), CSS 100%; supports HDR safety and efficacy	[58]
Achkar (2024)	France	Retrospective cohort, single institution	65	PDR	–	3 year DFS 74	NR	NR	Painful ulceration 20% (G2, resolved); urethral stenosis 22% (G2, 8% surgical); no G3 events; identified dose–toxicity link	85 (3 year)	3D PDR BT achieved high LC (~ 82%) and PPR 85% with moderate morbidity; urethral dose constraints > 82 Gy (D0.1 cc) predicted stenosis	[59]
Ka (2025)	France	Multicentric retrospective cohort	408	LDR, PDR, HDR	86	3 year LC ≈ 82	NR	NR	Acute ≥ G2 34% (G3 14%, G4 0.7%), late urethral stenosis 18%, painful necrosis 16%, 10% required surgery	85 (5 year)	Largest multicenter BT series confirms durable 5-yr LC (86%) and PPR (85%) with acceptable toxicity; circumcision reduced ≥ G2 toxicity; lymph node status strongest predictor of progression	[60]

*BT* Brachytherapy, *LDR* Low-dose-rate brachytherapy, *HDR* High-dose-rate brachytherapy, *PDR* Pulsed-dose-rate brachytherapy, *RFR* Recurrence-free rate, *LRFS* Local recurrence-free survival, *DFS* Disease-free survival, *CSS* Cancer-specific survival, *OS* Overall survival, *PPR* Penile preservation rate, *LC* Local control, *FU* Follow-up, *QoL* Quality of life, *IIEF* International Index of Erectile Function, *IIEF-5* Five-item version of the International Index of Erectile Function (erectile function score), IMGI International Male Genital Image Scale, *LUTS* Lower urinary tract symptoms, *IPSS* International Prostate Symptom Score, *CTCAE* Common Terminology Criteria for Adverse Events, *EQD2* Equivalent dose in 2 Gy fractions (biologically equivalent radiation dose), *ED* Erectile dysfunction, *NR* Not reported, *NA* Not applicable, *NS* Not specified, *SCC* Squamous cell carcinoma, *PP* Partial Penectomy

The 18 included studies encompass together a total of 1137 patients that received organ-sparing brachytherapy. Included studies originated primarily from European centers, along with contributions from Canada and India. The most common design was a retrospective, single-center cohort study.

### Variability of brachytherapy approaches

A basic numerical analysis reveals that out of the 18 studies, 13 studies [44–48, 50, 51, 54–59] presented cohorts using a single therapeutic approach and 5 studies [43, 49, 52, 53, 60] reported brachytherapy as a whole including patients that underwent either of the approaches without differentiation.

Details regarding the radiation source were not consistently reported across studies; however, the available contextual information allows a high degree of confidence in inferring the source used. Based on the radiation system used, Iridium-192 represents the standard for Nucletron systems employed for example by Makarewicz et al. [43], Rouscoff [46] or Kamsu-Kom [48]. Similarly, technical and dosimetry standards present in Canada employed by Garisto et al. [56] as well as in Europe in context studies such as the ones by Martz et al. [57], Achkar et al. [59] or Ka et al. [60] suggest Iridium-192 as the radiation source [61].

Based on the therapeutic approach, the included studies have been grouped by brachytherapy dose-rate techniques used: low-dose-rate (LDR), pulsed-dose-rate (PDR), and high-dose-rate (HDR). The findings and some approach characteristics of each study have been summarized accordingly in Tables 2, 3, and 4, respectively. To put in perspective, 16.67% of the studies have a cohort that only employs LDR or PDR therapy, 38.89% of which have a dedicated HDR cohort and 27.78% of which contain a mixed-treatment cohort. However, in terms of cohort sizes, the LDR-dedicated studies (Table 2) encompassed a total of 155 patients across three cohorts (13.6% of the 1137 patients in the review), PDR-dedicated studies (Table 3) included 105 patients across three cohorts (9.2% of total), and HDR-dedicated studies (Table 4) involved 187 patients across seven cohorts (16.4% of total).

**Table 2:** Studies overviewing LDR therapy

Author (year)	Number of participants	Dose (Gy)	5-yr LC (%)	PPR (%)	Major late toxicity	Key takeaway	References
Delaunay (2013)	47	60	84	66	Ulceration 21%, meatal stenosis 21%, pain 26%, mostly mild or moderate	LDR brachytherapy achieved high disease-specific and disease-free survival with moderate late toxicity and preserved erectile function in most patients	[45]
Córdoba (2016)	73	60	74	88 (5 year)	Dermatitis 12%, urinary trouble: 6%, stenosis 7%, sexual pain: 2%	Durable local control and high penile preservation with acceptable late toxicity in early-stage SCC	[50]
Garisto (2021)	35	-	80	69	Stenosis 16%, necrosis 8%, local infection 2%	Surgery achieved higher local control, but LDR brachytherapy preserved the organ in approximately 70% of cases with acceptable toxicity	[56]

*Gy* Gray, *LDR* low-dose-rate brachytherapy, *LC* local control, *PPR* penile preservation rate, *SCC* squamous cell carcinoma

**Table 3:** Studies overviewing PDR therapy

Author (year)	Number of participants	Dose (Gy)	3–5 year LC (%)	PPR (%)	Urethral stenosis (%)	Key takeaway	References
Kamsu-Kom (2015)	27	60	88	85	22	PDR brachytherapy achieved high local control with manageable toxicity in patients with penile SCC	[48]
Seibold (2016)	13	60	88	100	15	Long-term disease control with excellent organ preservation; most patients retained erectile function	[51]
Achkar (2024)	65	65	82	85	22	Modern image-guided PDR brachytherapy achieved strong LC and PPR with identifiable urethral dose–toxicity correlations (D0.1 cc > 82 Gy, D0.2 cc > 79 Gy associated with stenosis)	[59]

*PDR* pulsed-dose-rate brachytherapy, *LC* local control, *PPR* penile preservation rate, *SCC* squamous cell carcinoma, *Gy* Gray

**Table 4:** Summary of studies reporting high-dose-rate (HDR)

Author (year)	Number of participants	Technique	Dose/fractions	LC/RFR	PPR (%)	Notable toxicity	Key takeaway	References
Petera (2011)	10	Interstitial	54 Gy total (18 × 3 Gy, BID over 9 days)	–	100	Mild late telangiectasia only; no urethral stenosis or necrosis	Hyperfractionated HDR-BT (18 × 3 Gy BID) appears promising in selected penile carcinoma cases and warrants validation in larger prospective studies	[44]
Rouscoff (2014)	12	Interstitial	36 Gy (adjuvant) or 39 Gy (definitive) / 9 fractions over 5 days (BID)	5 year LRFS 83%; CSS 100%; OS 78%	92	Acute dermatitis (100%, G3 8%), telangiectasia 33%, urethral stenosis 9% (G3)	Conservative HDR technique provided favorable oncological and functional results; follow-up remains too short for definitive comparison with LDR/PDR outcomes	[46]
Sharma (2014)	14	Interstitial	42–51 Gy in 14–17 fractions, 3 Gy BID over 7–9 days	3 year LC ≈ 86%; 3 year OS 83%	93	Acute G3 dermatitis (healed), fibrosis 29%, no urethral stenosis/necrosis	HDR interstitial BT achieved excellent local control and organ preservation with maintained sexual function; longer follow-up needed to confirm equivalence with LDR/PDR	[47]
Kellas-Ślęczka (2019)	76	Interstitial (*n* = 70) + superficial (*n* = 6)	~ 42–57 Gy, BID 3–3.5 Gy	LC 66%; 1 year 85%, 3 year 72%	70 (5 year); 67 (10 year)	Pigmentation 36%, telangiectasia 21%, stenosis 1%; no G3–G4	Long-term outcomes comparable to LDR/PDR with minimal severe toxicity	[54]
Marbán-Orejas (2020)	15	Interstitial (*n* = 7) + surface mold (*n* = 8)	38.4–53 Gy/12–17 fx; Mold 40 Gy/10 fx	DFS ≈ 100% (1 relapse per group)	86–88	Interstitial: necrosis 43%, stenosis 43%; Mold: mostly skin changes	Both HDR modalities achieved excellent tumor control; adherence to homogeneity and urethral dose constraints is critical	[55]
Martz (2021)	29	Multicatheter interstitial	35 Gy (adjuvant) or 39 Gy (definitive) in 9 fx over 5 days	5 year LRFS 82%	79	G3 necrosis 10%, telangiectasia 17%, stenosis 7%	Strong 5-year control with stable urinary and sexual function; highlights importance of dose optimization to minimize necrosis	[57]
Pohanková (2022)	31	Interstitial, template-guided, CT-planned	54 Gy (18 × 3 Gy BID)	5 year 81%	5- ear 81; 10 year 62	Stenosis 7%, necrosis 3%	Prospective single-center: excellent control with very low necrosis	[58]

*BT* brachytherapy, *HDR* high-dose-rate, *LDR* low-dose-rate, *PDR* pulsed-dose-rate, *BID* twice daily, *LC* local control, RFR recurrence-free rate, *LRFS* local recurrence-free survival, *DFS* disease-free survival, *CSS* cancer-specific survival, *OS* overall survival, *PPR* penile preservation rate, *fx* fractions, *SCC* squamous cell carcinoma, *G* grade (toxicity), *Gy* Gray

The five studies [43, 49, 52, 53, 60] included mixed cohorts, in which patients were treated with more than one brachytherapy modality (LDR, PDR, and/or HDR). As outcomes were not stratified by technique, the combined cohort of 690 patients accounted for roughly 60% of the total treated population (Table 5).

**Table 5:** Summary of mixed-modality brachytherapy studies (LDR, PDR, HDR)

Author (year)	Number of participants	Techniques	Dose/fractions	LC / RFR	PPR (%)	Notable toxicity	Key takeaway	References
Makarewicz (2010)	33	HDR (48–54 Gy BID) and PDR (60 Gy/6 days) interstitial BT	HDR: 48–54 Gy; PDR: 60 Gy over 6 days	5 year LRC 79%; DFS 75%	≈ 85	Urethritis 30%, telangiectasia 15%, necrosis 9%*	Mixed HDR/PDR series demonstrated good 5 year control and high preservation with acceptable morbidity	[43]
Pimenta (2015)	25	LDR 56%, PDR 40%, HDR 4% interstitial BT	Median 60 Gy (Ir-192); dose rate varied by era	LC ≈ 92% (median FU 9.2 yr)	86	Urethral stenosis 43%, telangiectasia 48%, fibrosis 9%	Long-term control excellent across modalities; stenosis frequent in LDR/PDR	[49]
Escande (2017)	201	LDR (*n* = 166) and PDR (*n* = 35) interstitial BT	Median 65 Gy (0.4–0.5 Gy/h PDR)	5 year LC 82%	85	Meatal stenosis 26%, ulceration 21%, fibrosis 30%	Large single-center cohort confirmed durable 5 year control; toxicity correlated with dose > 65 Gy and > 2 planes	[52]
Gambachidze (2017)	23	LDR (11) and PDR (12) interstitial BT	Median 65 Gy (0.4 Gy/h; EQD2 ≈ 63 Gy)	-	100% (all disease-free ≥ 3 yrs)	Painful ulceration 26%, urethral dilation 30%, necrosis 26%	Functional study: mild–moderate urinary/sexual impact; median QoL 80 / 100; supports BT as first-line organ-sparing therapy	[53]
Ka (2025)	408	LDR 61.5%, PDR 29.2%, HDR 9.3% interstitial BT	LDR ≈ 65 Gy; PDR ≈ 60 Gy; HDR 36–39 Gy (CT-guided)	5 year LC 86%	85	Acute ≥ G2 34%, late stenosis 18%, necrosis 16%	Largest multicenter cohort confirming durable 5 year LC/PPR across all dose rates; circumcision reduced acute ≥ G2 toxicity	[60]

*BT* brachytherapy, *LDR* low-dose-rate, *HDR* high-dose-rate, *PDR* pulsed-dose-rate, *LC* local control, *LRC* locoregional control, *RFR* recurrence-free rate, *DFS* disease-free survival, *PPR* penile preservation rate, *FU* follow-up, *QoL* quality of life, *Gy* Gray, *EQD2* equivalent dose in 2 Gy fractions, *G* grade (toxicity)

### Oncological outcomes

Local relapses were generally amenable to successful salvage treatment, with Escande et al. [52] reporting a 77.4% salvage success rate for isolated local recurrence in a large cohort *n* = 201. This supports previous findings on smaller cohorts by Makarewicz et al. [43] who showed 80% surgical control after local/locoregional failure in HDR/PDR (*n* = 33) or Kamsu-Kom et al. [48] (*n* = 27) with salvage partial penectomy performed in patients with local recurrence (15%). Finally, in chronological order, another small cohort by Pohanková et al. [58] (*n* = 31) shows all post-salvage patients locally controlled in long follow-up after HDR.

Cancer-specific survival (CSS) was variably documented across the 18 studies with 13 offering explicit or implicit cancer-specific survival findings [45, 46, 49–58]. Condensing their findings leaves us with nine studies which provided explicit CSS rates. Five-year CSS was reported with results ranging from 85.0% [54] up to 100% [46, 55, 58]. It is worth observing that the lower end CSS cohort consisted of 9% T3 and ~ 12% Tx staged patients. Other studies’ CSS results showed 95% [50], 91.3% [49], 88% [57], and 87.6% [45] five-year rates. Ten-year CSS rates were reported as 100% [58], 95% [50], 91.3% [49], 88% [56], and 77.8% [54]. Particularly in terms of follow-up period, one study reported 100% CSS for both an interstitial HDR cohort (median follow-up 90 months) and a mold HDR cohort (median follow-up 27 months) [55].

### Functional and patient-reported outcomes

Regarding penile preservation, organ-preserving potential has been shown across the 18 studies, with penile preservation rates (PPR) exceeding 80% at 5 years in larger cohorts [52, 60]. Explicit PPR ranged from 66% [45] to 100% [44, 51], with 5 year rates of 85% in multicenter series [52, 60] and 79.3% in HDR-focused groups [57].

Six studies showed that particularly for T1–T2 stage disease, brachytherapy represents a viable organ-sparing therapy option with reported rates of 87.5% and 86% respectively [55], 86.1% at 5 years [49]; 85% at 5 years [52], 85% (3 year penectomy-free survival) in a cohort with 92% T1–T2 tumors [59]; and 85% (5 year penectomy-free survival) in a cohort with 96.1% T1–T2 tumors [60].

Salvage penectomy was typically required for local failure or necrosis (15–27% of cases) but conservative management achieved high preservation, particularly in early-stage tumors [43, 50, 52, 54, 56, 57, 59, 60].

Sexual function was preserved in most sexually active patients, with IIEF-5 scores showing no significant decline in prospective assessments such as median 16 pre- to 13 at 24 months as shown by Rouscoff et al. [46] or > 21 in 91% post-treatment as reported by Sharma et al. [47]. Ad hoc evaluations indicated unchanged erectile function, inter-course frequency, and satisfaction in 80–90% [43, 44, 55], with 58–85% maintaining activity comparable to baseline [45, 53, 58]. Urinary function remained stable overall, with IPSS unchanged in monitored series [46, 57]. Complications such as meatal/urethral stenosis affected 7–43% [49, 50, 57] (requiring dilatation in 13–26% [52, 53]), with urinary incontinence reported less frequently [49]. Urethritis represented another minor but common (30%) complication [43, 53]. Cosmetic outcomes were good to excellent in smaller series, with telangiectasia (15–48%) [43, 49], fibrosis, or pigmentation being well-tolerated and with minimal psychosexual impact [43, 47, 55].

Quality of life assessment data were sparse, with one study reporting a median QoL score of 80 impacted primarily by pain, as shown by Gambachidze et al. [53]. Sharma et al. reported for example that “hyperpigmented patches “had no psychosexual impact” [47]. Kellas-Sleczka et al. reported that “telangiectasia and pigmentation changes were well tolerated by all patients” [54]. Similar reports were noted by Makarewicz et al. and Marban et al. regarding telangiectasia [43, 55].

## Discussions

To facilitate transparent evaluation of the evidence, we applied a hierarchical assessment considering study design (prospective versus retrospective), sample size, follow-up duration, and completeness of outcome reporting. Single-center retrospective series with small sample sizes (< 30 patients) and short follow-up (< 3 years) were considered low certainty evidence. Larger single-center series (> 50 patients) with longer follow-up (> 5 years) or multicenter studies were considered moderate certainty. Prospective studies and multicenter collaborations with standardized protocols were weighted most heavily. Throughout this discussion, we explicitly characterise evidence certainty to aid readers in assessing the strength of our conclusions.

### Comparative efficacy across brachytherapy modalities

In this systematic review of 18 studies on brachytherapy for localized penile squamous cell carcinoma, brachytherapy with LDR, PDR, and HDR techniques approaches demonstrate high cancer-specific survival (CSS) rates of 85–100% at 5 years with regards to early-stage disease [46, 54, 55, 58]. Noteworthy, this is achieved with high rates of organ preservation (often 85% or higher), supporting it as an effective primary treatment for T1–T2 penile cancer [49, 52, 55, 59, 60].

One of the research questions is which option is the better therapeutic approach. A direct and "fair" comparison between LDR, PDR, and HDR brachytherapy approaches is not feasible due to simultaneous differences in study populations, variation in dosimetry and technique, and evolution of technology and planning across the years. Besides the first set of characteristics which are difficult to modify, heterogeneous outcome reporting and small sample sizes across many of the included studies, represent drawbacks, mainly stemming from the rarity of the disease [62]. Bearing all this in mind, the following comparative overview of the results of the techniques by no means provides a definitive answer to the question of which approach is superior.

Based on low to moderate certainty evidence from predominantly retrospective single-center studies, outcomes were broadly comparable across modalities. Among studies employing a single brachytherapy modality, 5 year CSS rates ranged between 87.6 and 95% for LDR cohorts [45, 50, 56], 3–10 year CSS ranged between 84.6 and 100% for PDR [48, 51, 59] and 5–10 year CSS of 77.8–100% for HDR cohorts.

While oncologic control was good, specific LDR cohorts reported lower penile preservation, with amputation-free or preservation rates at 5 years or longer ranging from 66 to 77% [45, 50, 56]. This was driven by both local recurrence (20–34%) [45, 56] and a notable rate of toxicity-driven penectomy for necrosis (6.8–11.4%) [50, 56]. The PDR-only cohorts demonstrated consistently good preservation outcomes, with reported rates of 85–100% [48, 51, 59], alongside good 3–5 year local control (82–88%) [48, 59]. Seibold et al. in particular, noted 0% mutilating salvage surgery at a 54 month follow-up [51]. The HDR-only studies presented the most variable results. While several smaller, prospective series reported excellent outcomes with > 90% preservation [44, 46, 47], larger series or those with longer follow-up [54, 58] reported more modest 5–10 year local control (65.6–80.7%) and preservation rates (69.5–71%).

It is noteworthy that some of the studies [43, 49, 52, 53, 60], which reported high 5 year preservation rates of around 85% [52, 60], utilised mixed-modality cohorts, preventing a clear isolation of the superior technique. Moderate certainty evidence from the largest multicenter series (*n* = 408) reported 5 year local control of 86% and penile preservation of 85% across all three modalities [60], suggesting that when delivered by experienced centers, all approaches achieve similar outcomes. The evidence suggests that all three modalities are viable, with slightly better outcomes in the long run (more than 5 year follow-up) for the newer PDR and HDR techniques. This corresponds to previous comparisons which included older studies of Martz et al. and Pohankova et al. [57, 58]. However, substantial heterogeneity in dosimetry, fractionation, and technique prevents definitive conclusions about superiority of any single modality. To provide a structured overview of comparative outcomes across dose-rate modalities, Table 6 synthesizes oncologic efficacy, preservation rates, toxicity, and evidence certainty.

**Table 6:** Evidence synthesis: comparative outcomes by brachytherapy modality

Modality	Number of studies (Total *n*)	Predominant study design	5 year CSS range	5-year penile preservation rate (PPR)	Major toxicity (Urethral Stenosis)	Evidence certainty
LDR-only	3 (*n* = 155)	Retrospective single-center	87.6–95% [45, 50, 56]	66–77% [45, 50, 56]	7–21%	Low–Moderate
PDR-only	3 (*n* = 105)	Retrospective single-center (one with 3D imaging)	84.6–100% [48, 51, 59]	85–100% [49, 52, 60]	15–22%	Low–Moderate
HDR-only	7 (*n* = 187)	Mostly retrospective; one prospective	77.8–100% [44, 46, 47, 54, 55, 57, 58]	69.5–93% [44, 46, 47, 54, 55, 57, 58]	1–43% (wide variation)	Low–Moderate
Mixed cohorts	5 (*n* = 690)	Retrospective; includes largest multicenter study (*n* = 408)	85–100%* [52, 60]	85–86% [52, 60]	18–43%	Moderate

*CSS* varied across cohorts

*Largest multicenter study reported 5-year local control of 86% across modalities

### Brachytherapy versus surgical penectomy: evidence and trade-offs

In terms of comparison between brachytherapy and partial or total penectomy as a first treatment approach, brachytherapy performed closely in terms of survival, with the upside of psychological and physiological advantages of organ preservation. As Hasan et al. show in an older meta-analysis including more than 1000 patients, brachytherapy achieves 74–86% 5 year local control versus 84% for penectomy, but with no overall survival difference (due to effective salvage in 77–80% of brachytherapy failures) [63]. PPR exceeds 80% at 5 years with brachytherapy, versus the obvious 0% with penectomy providing a convincing argument [52, 60]. This quantifies the oncologic versus functional trade-off: brachytherapy accepts approximately 5–10% lower local control in exchange for 80–85% organ preservation, with equivalent overall survival due to highly effective salvage surgery. Penectomy ensures superior local control in advanced cases but at the cost of mutilation, clearing brachytherapy’s role in T1–T2 disease, hence the importance of early diagnosis and case selection. In this regard, patient selection should also account for comorbidities; for instance, the poorer outcomes noted in diabetic patients [50] suggest that vascular health may be a critical determinant in balancing the risk of late-term necrosis against the benefits of preservation.

Beyond radical surgery, multidisciplinary strategies often evaluate conservative surgical options like glansectomy or wide local excision to mitigate functional morbidity [13, 14]. As noted in the clinical landscape of the included studies, while surgical sparing techniques provide local control for superficial T1a disease, brachytherapy offers a clear advantage for T1b–T2 tumors. In these cases, achieving wide negative margins through surgery alone would frequently result in significant anatomical deformity or glans volume loss, whereas brachytherapy provides volumetric coverage while maintaining the structural integrity of the organ [46, 54]. Additionally, the potential for combined approaches—such as utilising brachytherapy in an adjuvant setting after conservative excision with close or positive margins—represents an emerging strategy to maximise local control while upholding the primary objective of organ preservation.

Psychologically, brachytherapy yields superior QoL, lower anxiety/depression (vs. high post-penectomy rates), and better sexual/psychosexual adjustment [53, 55, 66]. Evidence from included studies demonstrates that 58–85% of patients maintain sexual activity following brachytherapy [45, 53, 58], with IIEF-5 scores showing no significant decline in prospective assessments [46, 47, 57]. However, this functional preservation comes at the cost of notable late toxicity, particularly urethral stenosis (7–43% across studies) [49, 50, 57, 59, 60], which often requires intervention through dilatation or self-catheterization [52, 53].

### Resource requirements and implementation considerations

The multiple techniques (LDR, PDR, HDR) and the complexity of planning and implantation illustrated in the included studies underscore that this is a resource-intensive treatment option. Penile brachytherapy is feasible in specialised centers with interstitial expertise, requiring hypodermic needles/applicators, CT/MRI-based dosimetry, and after loaders (HDR/PDR). Cost data are sparse for penile cancer, so we use prostate analogs which suggest brachytherapy to be cheaper by around 20% than prostatectomy when accounting for associated costs [64, 65]. The necessity for multidisciplinary teams with urologists, radiation oncologists, physicists, and psychologists as well as auxiliary personnel and even infrastructure barriers, limits adoption in low-resource settings.

### Limitations of our study

This systematic review has several limitations that must be acknowledged. The evidence base is composed of majorly single-institution, non-randomized studies, the majority of which are retrospective. This design is highly susceptible to selection bias (e.g., in which patients were offered brachytherapy versus surgery) and limits the overall strength of the conclusions.

Second, there was substantial heterogeneity across the 18 studies. This was most prominent in the treatment regimens (mixed or single approaches among the 3), as well as variable total doses and fractionation schedules. This variability prevents a formal pooled quantitative analysis (meta-analysis) and makes it hard to definitively determine the superiority of any single modality; the observed benefits may be context-dependent on institutional expertise.

Third, reporting of outcomes was inconsistent. While oncologic endpoints (e.g., survival, local control) were clearly defined, functional and safety data were not. Standardized, validated instruments, such as IIEF-5, IPSS, and QoL scales, were rare [53, 55], making an objective assessment of sexual and urinary function difficult. Similarly, the definition and grading of key adverse effects, such as urethral stenosis and necrosis, were inconsistent, limiting the ability to ascertain their true incidence and severity. These reporting gaps represent areas of uncertainty where current evidence cannot provide definitive guidance, and clinical judgment based on individual patient factors must guide treatment selection.

Finally, the included studies had highly variable follow-up durations (from 20 months [44] to over 10 years [52]) and as a result, studies with shorter follow-up may underestimate these risks. As with most systematic reviews, the possibility of publication bias—meaning that studies with negative or unfavorable results are less likely to be published—should be mentioned. The geographic concentration of studies in European centers, with limited representation from other regions, may also limit generalizability of findings.

### Future research directions

Based on the limitations identified in the 18 studies, future research objectives must prioritize generating higher-quality, comparative data. To strengthen the evidence base, future research should advance beyond single-center retrospective series and prioritize the development of prospective, multicenter randomized controlled trials (RCTs) directly comparing brachytherapy modalities. While the rarity of penile cancer makes traditional RCTs challenging, a more immediately feasible goal is the standardization of outcome reporting through international registries. Such registries would facilitate the pooling of data from multiple centers to answer unanswered clinical questions, such as the optimal total dose and fractionation for T2b–T3 lesions where the risk of necrosis is the highest.

From a technological perspective, emerging advances in MRI-guided adaptive brachytherapy planning promise real-time dose optimization based on daily anatomy, which is a critical technological advance for reducing urethral toxicity while maintaining high efficacy [59, 60]. Furthermore, the integration of molecular and HPV biomarkers for treatment selection represents a major frontier; the identification of radiosensitive phenotypes could enable personalized dose de-escalation, significantly improving the safety profile of organ-sparing radiotherapy [27, 30]. Additionally, the development of consensus guidelines for dose–volume constraints to organs at risk—particularly the urethra—and the establishment of standardized functional outcome reporting through core outcome sets are essential. These efforts would transform penile brachytherapy from an institution-specific expertise into a standardized, evidence-based global practice.

## Conclusions

In conclusion, the available evidence, primarily from retrospective single-center and recent multicenter series, suggests that penile brachytherapy—across LDR, PDR, and HDR techniques—is a viable organ-preserving treatment for localized T1–T2 penile cancer. These modalities demonstrate favorable 5-year cancer-specific survival (85–100%) and maintain high rates of organ preservation, typically around 85%. While local failure rates are higher than those associated with radical surgery, the high success rate of surgical salvage (77–100%) ensures that long-term survival is not compromised, providing a significant quality-of-life advantage in terms of sexual function and psychological well-being.

However, these findings must be interpreted with caution due to the high level of study heterogeneity and the lack of prospective, randomized comparisons. The primary trade-off for organ preservation remains a notable incidence of late toxicities, such as urethral stenosis (7–43%), which requires ongoing clinical vigilance. Significant knowledge gaps persist regarding the optimal dose-fractionation schedules and the standardized use of patient-reported outcome measures. Future research utilizing MRI-guided adaptive planning and molecular biomarkers is necessary to transition from institutional expertise to a standardized, evidence-based global practice.

## Supplementary Information

Below is the link to the electronic supplementary material.

Supplementary file1 (DOCX 1906 KB)

## Data Availability

No datasets were generated or analyzed during the current study.
