# Supplementary material for: Organ-sparing radiotherapy in penile cancer: therapeutic approaches
Source: Int Urol Nephrol. 2026 Apr 17;58(9):3419–38. doi: 10.1007/s11255-026-05135-y (PMC13506616; doi:10.1007/s11255-026-05135-y)
Supplement: Supplementary file 1 [file 11255_2026_5135_MOESM1_ESM.docx]

**Organ-Sparing Radiotherapy in Penile Cancer: Therapeutic Approaches – Supplementary Materials**

**Supplemental Table S1.** Quality Assessment of Included Studies. Y = yes; N = no; CD = cannot determine; NA = not applicable; NR = not reported; RC = Retrospective cohort, PC= Prospective Cohort; CS = Case Series.

| **Author**  **(Year)** | **Study Design** | 1 | 2 | 3 | 4 | 5 | 6 | 7 | 8 | 9 | 10 | 11 | 12 | 13 | 14 | Quality |
| --- | --- | --- | --- | --- | --- | --- | --- | --- | --- | --- | --- | --- | --- | --- | --- | --- |
| Makarewicz (2010) | RC | Y | Y | NR | Y | N | Y | Y | CD | Y | N | Y | NR | N | N | Fair |
| Petera  (2011) | CS | Y | Unclear | Y | Y | Y | Y | Y | Y | - | - | - | - | - | - | Included |
| Delaunay (2014) | RC | Y | Y | Y | Y | N | Y | Y | CD | Y | N | Y | NR | CD | N | Good |
| Rouscoff (2014) | RC | Y | Y | NR | Y | N | Y | CD | N | Y | N | Y | NR | Y | N | Fair |
| Sharma  (2014) | PC | Y | Y | NR | Y | N | Y | CD | CD | Y | N | Y | NR | Y | N | Fair |
| Kamsu-Kom (2015) | RC | Y | Y | CD | Y | N | Y | Y | CD | Y | N | Y | NR | Y | N | Good |
| Pimenta (2015) | RC | Y | Y | CD | Y | N | Y | Y | Y | Y | N | Y | NR | Y | N | Good |
| Córdoba (2016) | RC | Y | Y | Y | Y | N | Y | Y | CD | Y | N | Y | NR | Y | Y | Good |
| Seibold  (2016) | RC | Y | Y | CD | Y | N | Y | Y | CD | Y | N | Y | NR | Y | N | Fair |
| Escande (2017) | RC | Y | Y | Y | Y | N | Y | Y | Y | Y | N | Y | NR | Y | Y | Good |
| Gambachidze (2017) | RC | Y | Y | Y | Y | Y | N | Y | Y | Y | N | Y | NR | N | CD | Fair |
| Kellas-Ślęczka (2019) | RC | Y | Y | Y | Y | N | Y | Y | Y | Y | N | Y | NR | Y | N | Good |
| Marbán-Orejas  (2020) | RC | Y | Y | Y | Y | N | Y | Y | Y | Y | N | Y | NR | Y | N | Good |
| Garisto  (2021) | RC | Y | Y | Y | Y | N | Y | Y | Y | Y | N | Y | NR | Y | N | Good |
| Martz  (2021) | RC | Y | Y | Y | Y | N | Y | Y | Y | Y | N | Y | NR | Y | CD | Good |
| Pohanková (2022) | PC | Y | Y | Y | Y | N | Y | Y | CD | Y | N | Y | NR | Y | N | Good |
| Achkar  (2024) | RC | Y | Y | Y | Y | N | Y | Y | Y | Y | N | Y | NR | Y | Y | Good |
| Ka  (2025) | RC | Y | Y | Y | Y | N | Y | Y | Y | Y | N | Y | NR | Y | Y | Good |

**Supplemental Figure S1.** Risk of bias assessment of non-randomised studies: Traffic-light plot.

**Supplemental Figure S2.** Risk of bias assessment of non-randomised studies: Summary plot
